# Updated treatment guidelines for drug-resistant TB: how safe are clofazimine-based regimens?

**DOI:** 10.5588/ijtldopen.24.0490

**Published:** 2024-11-01

**Authors:** E. Pontali, M. Raviglione

**Affiliations:** ^1^ Department of Infectious Diseases, Galliera Hospital, Genoa, Italy;; ^2^ MACH Centre, Università di Milano, Milan, Italy.

**Keywords:** QTc prolongation, Cfz, tuberculosis, MDR-TB, XDR-TB, aDSM

## Abstract

In June 2024, WHO released ‘Key updates to the treatment of drug-resistant tuberculosis: rapid communication’, after the preliminary publication of results from two clinical trials: ‘BEAT-Tuberculosis’ and ‘endTB’. All proposed regimens include clofazimine (Cfz). However, a recent paper has reported a high incidence of QTc prolongation among patients receiving Cfz-based treatment for multidrug-resistant TB in Taiwan. Here, we discuss the cardiac safety of Cfz and the role of active drug safety monitoring at the programme level in collecting information on this issue.

Over the past decades, increasing evidence has emerged on new regimens to treat rifampicin-resistant/multidrug-resistant/extensively drug-resistant-TB (RR/MDR/XDR-TB). Three new anti-TB drugs (effective also on RR/MDR/XDR TB) have been developed and registered: bedaquiline (Bdq, B), delamanid (Dlm, D) and pretomanid (Pa). Various regimens combining old and new drugs have been tested; some are more effective and better tolerated than previous second-line medicines. This evidence has been the basis for the WHO recommendations and guidelines for the treatment of RR/MDR/XDR-TB^1^ (Table 1). The WHO has continuously updated recommendations and guidelines, recommending appropriate combinations of drugs and determining the duration of each regimen. Since the first complex and toxic multidrug regimens lasting for up to 18–24 months, new regimens, including a reduced number of oral drugs and lasting for just 6–9 months, have been recommended for the treatment of RR/MDR/XDR-TB.^1^ In June 2024, the WHO released ‘Key updates to the treatment of drug-resistant TB. Rapid communication’, before the complete version of the guidelines.^2^ This rapid communication was issued after the preliminary publication of results from the BEAT-Tuberculosis clinical study and the endTB clinical trial (https://conf2023.theunion.org/programme/). Both trials used combinations that included clofazimine (Cfz, C). The BEAT-Tuberculosis clinical trial compared a new 6-month regimen composed of Bdq, Dlm, linezolid (Lzd, L) (600 mg daily), levofloxacin (Lfx), and Cfz (known as the BdqDlmLzdLfxCfz or the BDLLfxC regimen), with the currently WHO-recommended regimens in patients with MDR/RR-TB or pre-XDR-TB. The endTB clinical trial compared the use of five different 9-month, all-oral regimens comprising different combinations of Bdq, Lfx or moxifloxacin (Mfx, M), Lzd, Cfz, Dlm, and pyrazinamide (PZA, Z): BdqLzdMfxPza, BdqLzdLfxCfzPza, BdqDlmLzdLfxPza, DlmCfzLzdLfxPza, and DlmCfzMfxPza (known respectively also as BLMZ, BLLfxCZ, BDLLfxZ, DCLLfxZ and DCMZ) in MDR/RR-TB patients without resistance to fluoroquinolones (FQs) and with no previous exposure to second-line drugs with the WHO-recommended longer (≥18 months) regimens.

**Table 1.** WHO-recommended regimens for the treatment of RR/MDR/XDR-TB.

Regimen name	Drugs composing the regimen	Duration of treatment (months)	Indication/notes
BPaLM	Bdq, Pa, Lzd (600 mg), Mfx	6	RR/MDR-TB patients aged ≥15 years; Most recommend regimen
BPaL	Bdq, Pa, Lzd (600 mg)	6	RR/MD-TB patients aged ≥15 years; documented resistance to FQs Most recommend regimen
Shorter, all-oral, Bdq-containing regimen	Bdq (4 months; up to 6 months), Lfx or Mfx, Eto or Pto, Emb, Inh^H^, Pza, Cfz, followed by 5 months of Lfx or Mfx, Emb, Pza, Cfz	9–12	Lzd instead of Eto may be used in pregnant women Preferred over longer regimen Can be used in children
Longer regimen	All three Group A agents (Bdq, Lzd, Lfx or Mfx) and at least one Group B agent (Cfz or cycloserine/terizidone) should be included If only one or two Group A agents are used, both Group B agents are to be included If the regimen cannot be composed with agents from Groups A and B alone, Group C agents (Emb, Dlm, Pza, imipenem–cilastatin or meropenem, amikacin (or streptomycin), Eto or Pto, and p-aminosalicylic acid) are added	18–20 (modifiable based on response)	Obsolete; it contains injectables
BEAT	Bdq, Dlm, Lzd (600 mg), Lfx, and Cfz (BdqDlmLzdLfxCfz, also known as BDLLfxC)	6	
endTB	Bdq, Lfx or Mfx, Lzd, Cfz, Dlm, and Pza (BdqLzdMfxPza, BdqLzdLfxCfzPza, BdqDlmLzdLfxPza, DlmCfzLzdLfxPza, and DlmCfzMfxPza, also known as respectively BLMZ, BLLfxCZ, BDLLfxZ, DCLLfxZ, and DCMZ)	9	No resistance to FQs

RR/MDR/XDR-TB = rifampin-resistant/multi-drug resistant/extensively drug-resistant-TB; Bdq = bedaquiline; Pa = pretomanid; Lzd = linezolid; FQ = fluoroquinolone; Mfx = moxifloxacin; Lfx = levofloxacin; Eto = ethionamide; Pto = prothionamide; Emb = ethambutol; Inh^H^ = high-dose isoniazid; Pza = pyrazinamide; Cfz = clofazimine; Dlm = delamanid.

However, shortly after the release of this rapid communication, Lin et al.^3^ reported a worrying observation on the incidence and intensity of Cfz-induced QT prolongation among RR-TB patients in Taiwan. Several new WHO-recommended regimens include Cfz as a key drug, which can be given as an alternative to an FQ or with Lfx or Mfx.^2,4^ The toxicity of Cfz is well known, but it is generally considered a safe drug.^5–9^ However, its specific potential for cardiac toxicity has not been prospectively well studied. Its effect on the heart resides in the potential for QT prolongation. Several other modern anti-TB drugs, such as Bdq, FQs, Dlm, and Pa, share this effect on QT.^10–12^ When composing anti-TB regimens to treat RR/MDR/XDR-TB and one or more of these drugs are included, it is difficult to predict the combined effect on QT for each patient. The QT interval on the electrocardiogram (ECG) corresponds to the electrical depolarisation-repolarisation of ventricles. It is measured from the beginning of the Q wave until the end of the T wave, and this measurement must be corrected (QTc) according to the heart rate. Two formulas exist for optimal correction: Fridericia’s and Framingham’s.^13^ Fredericia’s correction is recommended for RR/MDR/XDR-TB patients.^11^ The normal QTc duration is <450 ms in males and <470 ms in females. However, the QTc can vary by up to 75 ms in the same person during the day; thus, if at screening, a QTc >500 ms is detected, it is advisable to repeat the ECG within 24 h.^14^ Prolongation of the QTc interval beyond 500 ms is a well-known risk factor for ‘*torsade de pointes*’ (TdP), a form of polymorphic ventricular tachycardia. This arrhythmia usually terminates spontaneously, but sometimes it can occur in episodes with rapid succession and cause symptoms like palpitations, dizziness, syncope and, eventually, sudden death.^15–17^ In general, prolonged QTc is a good predictor of risk of TdP.^18,19^ Unfortunately, the correlation between length of the QTc interval and/or duration of QTc prolongation and TdP is not linear and depends on additional risk factors, which can be congenital and unmodifiable (e.g., being female, having long QT syndrome) or acquired (e.g., hypokalaemia, HIV infection, concomitant use of other QTc prolonging drugs).^11^ Nevertheless, only a small minority of people with prolonged QTc interval are predisposed to TdP. A literature review of 249 patients with TdP associated with non-cardiac QTc-prolonging drugs reported that apart from the drugs, 71% had at least two other risk factors.^20^ According to this review, the prevalence of sudden death directly attributable to TdP as a result of anti-TB drugs is likely <1%. The risk of TdP is most probably caused by the cumulative number of several risk factors in the same patient rather than the administration of specific drugs. Thus, it is important to carry out an initial screening combining a baseline electrocardiogram with some laboratory tests and the patient’s clinical history to assess individual risk.^11^

Relatively few studies have reported data on Cfz cardiac safety. There is wide variability in the incidence of QTc prolongation in the studied cohorts, and other factors are likely to be involved in QTc prolongation. Genetic factors may intervene in different populations. In fact, well-studied cohorts show large differences among the various study sites.^21^ The recent study in Taiwan^3^ showed a high incidence of this phenomenon associated with Cfz or high-dose Mfx, especially in the presence of other risk factors. An overview of cardiac safety from clinical trials or other studies is shown in Table 2. In general, while the WHO currently recommends several Cfz-based regimens, severe drug-related cardiac adverse events have rarely been described in TB patients, and those that have occurred are associated mainly with other additional risk factors. Clinicians should be trained to identify all situations likely to add to the risk of TdP to prevent these rare but potentially fatal events. They should 1) monitor and manage treatable existing conditions, including gastroenteritis, malnutrition and hypothyroidism; 2) carefully identify any ancillary medications being used by the patient (e.g., methadone, hydrochlorothiazide, furosemide, metoclopramide); and 3) inform patients to promptly seek medical care if they notice palpitations and feeling of faintness or if they had a syncope episode.

**Table 2.** Studies reporting on cardiac safety of clofazimine (Cfz).

Study	Year	Cfz patients *n*	Country	Cardiac safety	Associated factors
Lin et al.^3^	2024	321 (165 Cfz)	Taiwan	QT >500 ms: 18.4%	Cfz use, high dose Mfx, older age, comorbidities
Motta et al.^22^	2024	108	Belarus, Uzbekistan, South Africa	QT>500 ms: 0–9%	
Ali et al.^23^	2023	86	South Africa	No QT>500 ms	All children/adolescents <18 years
Li et al.^24^	2023	32	China	Cfz associated with the highest QT increase from the baseline	All patients receiving Bdq
Hughes et al.^21^	2022	282	Various: Ethiopia, Mongolia, South Africa, Vietnam	94 (33.3%) severe QT prolongation: 31 QT/QTcF ≥500 ms; 92 ≥60 ms QTcF increase from baseline QT >500 ms: 3.5–45.5%	Site: highest among Mongolians: 45.5%; all others: <12%
Brust et al.^25^	2021	179	South Africa	QT>500 ms: 4.5% ≥60 ms QTcF increase from baseline: 2.2%	All patients receiving Bdq
Isralls et al.^26^	2021	419	South Africa	QT >500 ms: 4.1%	All patients receiving Bdq
Borisov et al.^7^	2019	213	Several (26)	QT prolongation: 1.4% (3 patients from Italy; only one >500 ms)	Passive reporting; variable monitoring
Dalcolmo et al.^6^	2017	1,445	Brazil	No cardiac adverse events reported	Passive reporting

Bdq = bedaquiline; Mfx = moxifloxacin.

Large-scale evidence shows that new oral regimens are safer than initially reported, at least from a cardiac and QT prolongation perspective. Some populations and sub-populations may be at higher risk of QTc prolongation when using Cfz-based regimens, as shown by Lin et al.^3^ and Hughes et al.^21^ However, it is possible that these populations also suffer from specific predisposing factors, which may not be fully identified. Hopefully, future high-quality new evidence will help identify patients at greater risk, especially in large-scale programmatic settings. In this regard, reports from rigorous active drug safety monitoring (aDSM) activities at programme level, such as the one reported by Lin et al.,^3^ may represent the future sources of information. For now, Cfz should be used with caution in patients with multiple risk factors for QTc prolongation. A comprehensive and careful evaluation of patients is essential at baseline, and monitoring is recommended during treatment.
